# Pre- and in-hospital delays in the use of thrombolytic therapy for patients with acute ischemic stroke in rural and urban Egypt

**DOI:** 10.3389/fneur.2022.1070523

**Published:** 2023-01-20

**Authors:** Ahmed Nasreldein, Silke Walter, Khaled O. Mohamed, Ghaydaa Ahmed Shehata, Azza A. Ghali, Ahmed Dahshan, Klaus Faßbender, Foad Abd-Allah

**Affiliations:** ^1^Department of Neurology, Assiut University Hospitals, Assiut University, Asyut, Egypt; ^2^Department of Neurology, Saarland University Hospital, Homburg, Germany; ^3^Department of Neurology, Faculty of Medicine, Tanta University, Tanta, Egypt; ^4^Department of Neurology, Cairo University Hospitals, Cairo University, Cairo, Egypt

**Keywords:** pre-hospital, in-hospital, delays, thrombolytic therapy, urban, rural, Egypt

## Abstract

**Background:**

Reducing pre- and in-hospital delays plays an important role in increasing the rate of intravenous thrombolysis (IVT) in patients with acute ischemic stroke. In Egypt, the IVT rate has increased steadily but is still far away from an ideal rate.

**Aim:**

The study aimed to investigate the factors associated with pre- and in-hospital delays of IVT among patients with acute ischemic stroke coming from urban and rural communities.

**Methods:**

This prospective, multicenter, observational cohort study was conducted from January 2018 to January 2019. Patients with acute ischemic stroke, who did not receive IVT, were included in the study. Patients were recruited from three large university stroke centers in Egypt, Assiut (south of Egypt), Tanta (north of Egypt), both serving urban and rural patients, and the University Hospital in Cairo (capital city), only serving an urban community. All participants underwent the National Institutes of Health Stroke Scale and full neurological assessment, urgent laboratory investigations, and computed tomography or magnetic resonance imaging to confirm the stroke diagnosis. The patients were subjected to a structured questionnaire that was designed to determine the parameters and time metrics for the pre- and in-hospital delays among patients from rural and urban regions.

**Results:**

A total of 618 patients were included in the study, of which 364 patients (58.9%) lived in rural regions and 254 (41.1%) in urban regions. General demographic characteristics were similar between both groups. Approximately 73.3% of patients who arrived within the therapeutic time window were urban patients. The time from symptom onset till hospital arrival (onset to door time, ODT) was significantly longer among rural patients (738 ± 690 min) than urban patients (360 ± 342 min). Delayed onset to alarm time (OAT), initial misdiagnosis, and presentation to non-stroke-ready hospitals were the most common causes of pre-hospital delay and were significantly higher in rural patients. For patients arriving within the time window, the most common causes of in-hospital delays were prolonged laboratory investigations and imaging duration.

**Conclusion:**

The limited availability of stroke-ready hospitals in rural Egypt leads to delays in stroke management, with subsequent treatment inequality of rural patients with acute stroke.

## Introduction

Within the Middle East region, Egypt has the highest incidence of stroke (1). Epidemiological studies identified a prevalence of 963 per 100,000 Egyptians. The mean and median incidence rates were 187/100,000 and 181/100,000, respectively (2–6). Presentation of patients with acute ischemic stroke within the thrombolysis time window of 4.5 h after symptom onset is required to gain access to intravenous thrombolysis (IVT) treatment following standard stroke imaging assessment (7).

In 2016, IVT for acute ischemic stroke became government-covered and free of charge for treatment-eligible Egyptian patients with stroke (8). To deliver treatment, the number of stroke-ready hospitals has increased significantly in recent years, with currently 43 stroke units delivering the service (8). Despite the availability of IVT and increased numbers of stroke units in Egypt, nearly 94.2% of eligible patients with acute ischemic stroke (AIS) do not receive IVT (9). Patients living in rural communities are more at risk of arriving at the hospital too late for treatment. Moreover, a relevant number of patients presenting within the therapeutic treatment window do not receive IVT due to in-hospital delays. Thus far, the evidence available for IVT treatment delays of patients with AIS comes from stroke units serving urban areas only, and studies analyzing the factors responsible for pre- and in-hospital delays in Egyptian stroke centers are limited (9, 10). The aim of this study is to investigate the factors lying behind pre- and in-hospital delays in patients with stroke coming from both rural and urban communities in Egypt.

## Materials and methods

### Study design

**Figure 1:**
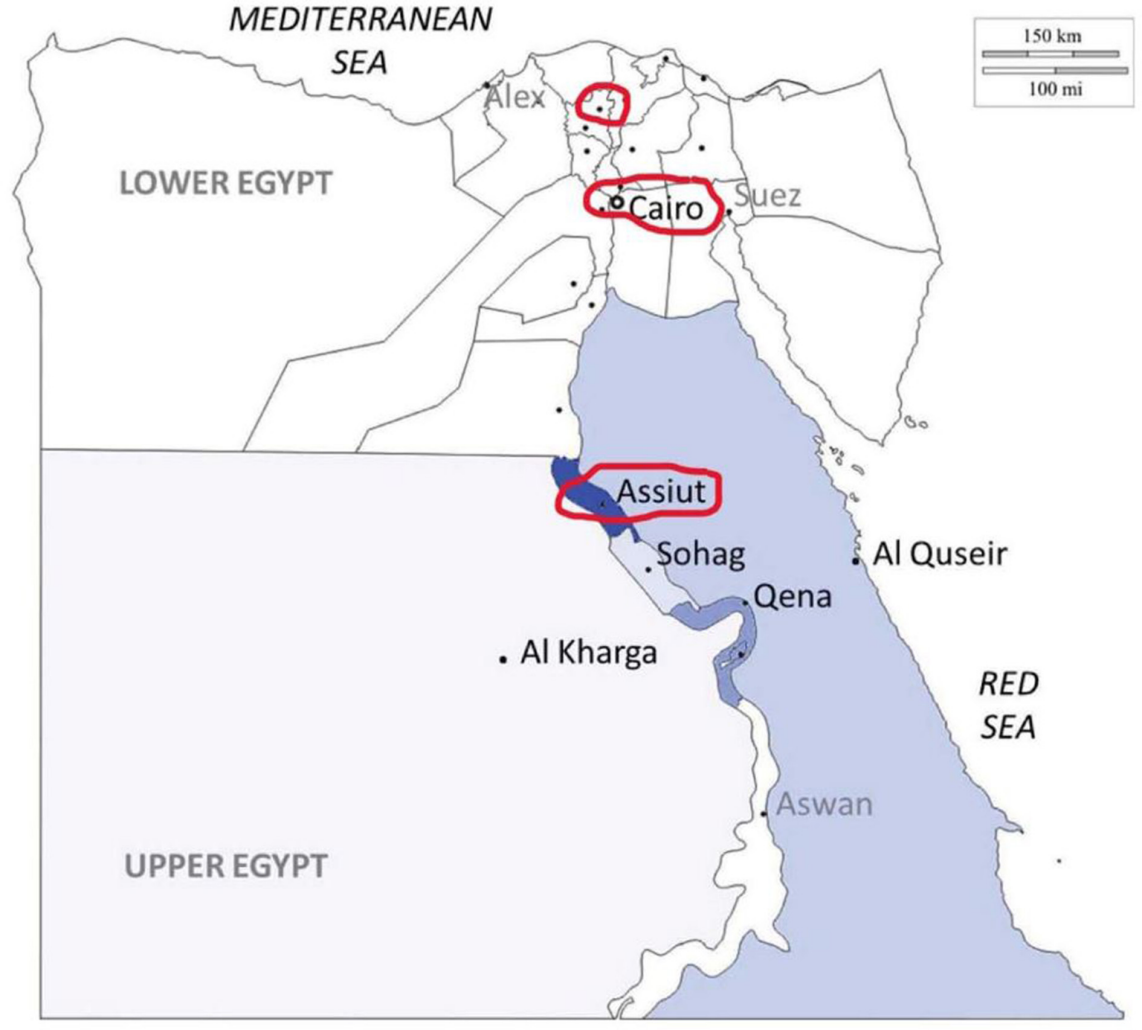
Egypt map showing the distribution of catchment areas.

From 1 January 2018 to 1 January 2019, we conducted a prospective, multicenter, open-label cohort study in three comprehensive stroke centers in Egypt. We compared stroke management of patients coming from rural to those coming from urban areas. Patients were recruited from the tertiary university hospitals of three different geographical regions in Egypt, which were Assiut University hospital, the largest tertiary stroke center in the south of Egypt, Tanta University hospital, in the north of Egypt, and Cairo University hospital (the capital of Egypt). Assuit and Tanta hospitals both serve rural and urban communities, while Cairo only serves an urban community (Figure 1). The Egyptian government classification system, which classifies all Egyptian regions into Governorates, districts (Markaz) (urban), and villages or satellites (rural), was used to allocate participants to the rural or urban groups (11). The study protocol was approved by the Ethical Committee of the Faculty of Medicine, Assuit University, Egypt, and followed the principles described in the Declaration of Helsinki.

### Patients' selection and stroke pathways

Consecutive patients with AIS presenting to the three comprehensive stroke centers participating in the study were recruited. To investigate the reasons for not receiving IVT, patients could only participate if they had not received IVT even though suffering from an IVT-eligible AIS. Rural patients with stroke came directly to these hospitals or were referred from other healthcare facilities, where IVT was not available. The enrolled patients fulfilled the following inclusion criteria: diagnosis of acute ischemic stroke, aged 18 years or older, not treated with IVT although eligible, and sought medical advice within the IVT time window either in places where IVT was not available or missed treatment because of in-hospital delay. Patients were excluded from participation if they suffered from a transient ischemic attack or had contraindications to IVT in their history (12).

### Data collection

**Figure 2:**
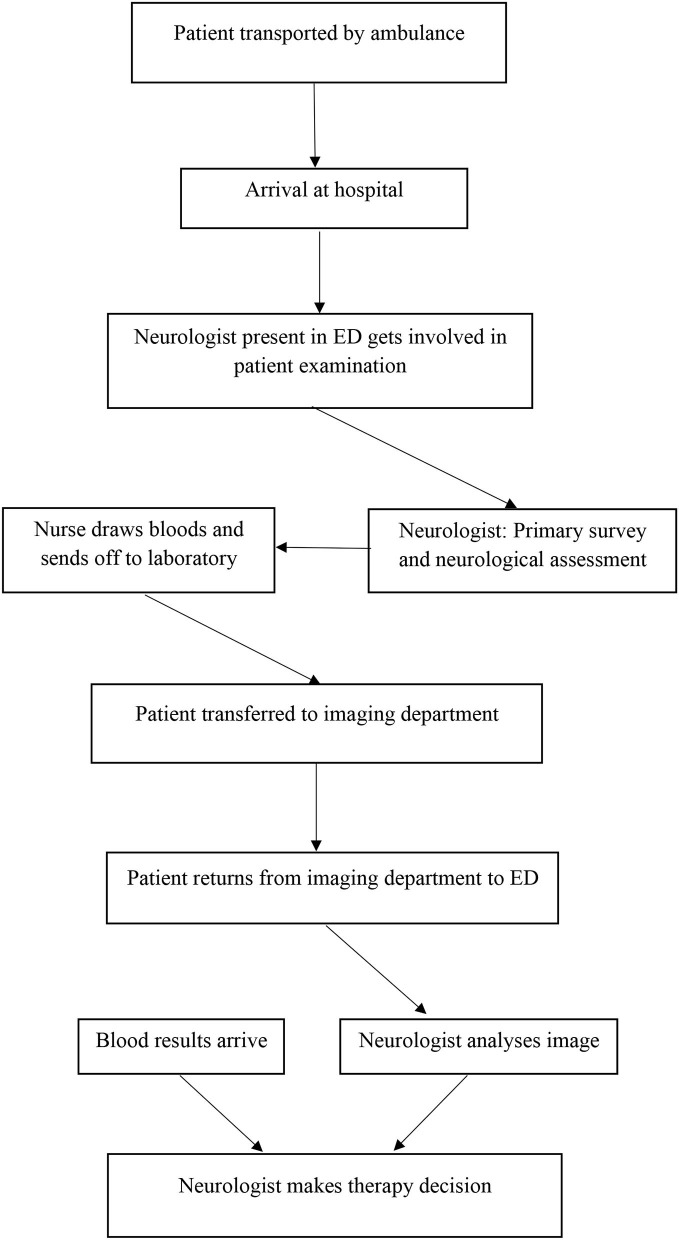
In-hospital workflow in the studied hospitals.

All patients were subjected to detailed history taking, neurological assessment, initial NIHSS assessment, computed tomography (CT) imaging, or magnetic resonance imaging (MRI) of the brain. Urgent laboratory investigations were performed for all patients in the emergency department (ED) (Figure 2). All information led to the patient's diagnosis. Information about the first diagnosis and the number of patients with correct acute stroke diagnoses was documented. Demographic data were collected for all participants and included age, gender, risk factors, previous medical history, and living distance to the next hospital, where IVT was available (13).

For all participants, the following management times were documented for later analysis: time of symptom onset, onset to alarm time (OAT), time of public ambulance response (in Egypt, the emergency medical service is run by the government and access to ambulances in an emergency is free to all Egyptians), time spent in referral from hospitals, where IVT was not available to the study participating centers, onset to door time (ODT), which is time from symptom-onset hospital door in minutes, time spent from hospital door to neurological examination, time spent in imaging (calculated from the time of sending the patient to imaging till imaging results), time spent till laboratory results, and time spent from arrival to the hospital to decision of management. In addition, we documented the type and the pathway of emergency patients, or their relatives used and the means of transport to the hospital.

As a safety outcome, the ability of physicians, ER doctors, EMS, or relatives to recognize the patient's symptoms as the stroke was calculated. The help-seeking behaviors of patients or their relatives and the most common diagnosis the patients had by initial assessment were investigated.

All data were collected by a structured questionnaire (Supplementary material).

### Statistical analysis

We analyzed the possibility of rural and urban variations in the pre- and in-hospital delays. Data obtained from this study were fed into an IBM-compatible computer. Descriptive statistics (mean (SD), numbers and percentages) were calculated using the Software Package for Social Sciences (SPSS, Inc, Chicago, Illinois) for Windows, version 25. Frequencies were noted and associations were determined using the Pearson chi-square test (X^2^ test). Also, results were analyzed using an independent-samples *t-*test that did not assume equal variances. The significance level was set at *p* < 0.05.

## Results

### Demographics

Of the total of 618 patients, 351 (56.8%) men and 267 (43.2%) women, were included in the study between January 2018 and January 2019. A total of 364 patients (58.9%) came from rural regions and 254 (41.1%) from urban regions. The mean age for patients living in the rural regions was 63.57 ± 11.51, and the mean for urban was 63.48 ± 11.96 (Table 1). Hypertension, dyslipidemia, DM, AF, valvular heart diseases, and smoking were the most common stroke risk factors among rural patients. DM, hypertension, dyslipidemia, smoking, AF, and valvular heart diseases were the most common risk factors among urban patients. There was no statistical difference in the risk factor distribution among people living in urban and rural regions.

**Table 1:** General demographics of the study population.

	**Rural** **(*n* = 364, 58.9%)**	**Urban** **(*n* = 254, 41.1%)**	***p*-value**
Male (*n*, %)	208 (57.1%)	143 (56.3%)	0.86
Female (*n*, %)	156 (42.9%)	111 (43.7%)	
Age (y) (Mean ± SD)	64 ±12	63 ± 12	0.92
NIHSS at admission (Mean ± SD)	9 ± 4	9 ± 4	0.49
mRS at admission (Mean ± SD)	3 ± 0.9	3 ± 0.9	0,18
Distance to hospital:			
<10 km	0	52 (20.5%)	
10–50 km	77 (21.2%)	166 (65.4%)	
51–100	194 (53.3%)	36 (14.2%)	
>100 km	93 (25.5 %)	0	
Risk factors distribution (*n*, %)			0.11
No risk factors	5 (1.4%)	3 (1.2%)	
Diabetes mellitus	73 (20 %)	77 (30.3%)	
Hypertension	104 (28.6%)	61 (24.0%)	
Ischemic heart disease	19 (5.2%)	10 (3.9%)	
Atrial fibrillation and valvular heart disease	38 (10.4%)	18 (7.1%)	
Dyslipidemia	81 (22.3%)	58 (22.9%)	
Smoking	37 (10.2%)	21 (8.3%)	
Vasculitis	7 (1.9 %)	6 (2.4%)	
Onset to alarm time (OAT) (in minutes) (Mean ± SD)	234 ± 192	172.2 ±135.6	<0.05
Time of ambulance response (in minutes) (Mean ± SD)	31.88 ± 5.98	26.86 ± 8.05	<0.05
Number of Patients referred from non-stroke ready hospitals	298 (81.9%)	40 (15.7%)	<0.05
Time spent (in minutes) for referral from non-stroke ready hospital to stroke ready hospital (Mean ± SD)	537 ± 684	534 ± 414	0.97

Data described as numbers and percentages or mean ± standard deviation according to need. Q square and independent *t-*tests were used.

### Pre-hospital time metrics and management pathways

The onset to alarm time was significantly longer among rural (234 ± 192 min) than urban patients with stroke (172.2 ± 135.6 min, *p* < 0.05). In addition, 85% of urban patients were living within 50 km of hospitals, where IVT was available in comparison to 21.2% only of our rural patients. Notably, 53.3% of rural patients live within 50–100 km and 25.5% live more than 100 km away from hospitals where IVT was available. This inequality contributed markedly to the pre-hospital delays among rural patients.

People in rural regions showed a higher tendency to contact emergency medical services than people living in urban regions. They also showed a higher tendency to seek medical advice from non-neurologists than the urban population (Table 2). In patients coming from rural areas, stroke diagnosis was significantly more often missed by the first treating physicians or examiners (68.7%) (Table 3). The time of ambulance response (from alarm to arrival at the patient's site) to the notification was also significantly longer among rural regions (31.88 ± 5.98 min in comparison to 26.86 ± 8.05 in urban regions).

**Table 2:** Type of help-seeking behaviors among the study population.

**Different types of contact for help (*n*, %)**	**Rural** **(*n* = 364, 58.9%)**	**Urban** **(*n* = 254, 41.1%)**	***p*-value**
Ambulance	97 (26.6 %)	51 (20.1%)	<0.05
General practitioner	64 (17.6 %)	31 (12.2%)	
Internal medicine physicians	59 (16.2 %)	39 (15.4%)	
Neurologists	30 (8.2 %)	50 (19.7%)	
Relatives, friends, neighbors	60 (16.5 %)	44 (17.3%)	
ENT (Ear, Nose, Throat physicians)	18 (4.9 %)	16 (6.3%)	
Ophthalmologists	13 (3.6 %)	4 (1.6%)	
Other medical specialties	18 (4.9 %)	17 (6.7%)	
Traditional healers	5 (1.4 %)	2 (0.8%)	

Patients contacted physicians in their private clinics, hospital outpatient clinics, and emergency rooms.

**Table 3:** Accuracy of stroke diagnosis variations among rural and urban regions.

**Initial diagnosis of the patients (*n*, %)**	**Rural** **(*n* = 364, 58.9%)**	**Urban** **(*n* = 254, 41.1%)**	***p*-value**
Recognize incident as stroke	114 (31.3 %)	111 (43.7%)	<0.05
Recognize the incident as non-stroke related	250 (68.7%)	143 (56.3%)	
Functional disorder	15 (4.1%)	12 (4.7%)	
Cardiovascular disorder	35 (9.6%)	9 (3.5%)	
Diabetic coma	34 (9.3 %)	12 (4.7%)	
ENT disorder	26 (7.1%)	22 (8.7%)	
Ophthalmic disorder	16 (4.4%)	7 (2.8%)	
GIT	2 (0.5%)	4 (1.6%)	
No diagnosis	53 (14.6%)	27 (10.6%)	
Coma of unclear cause	28 (7.7%)	32 (12.6%)	
Other diagnosis	41 (11.3%)	18 (7.1%)	

### In-hospital time metrics and management pathways

**Figure 3:**
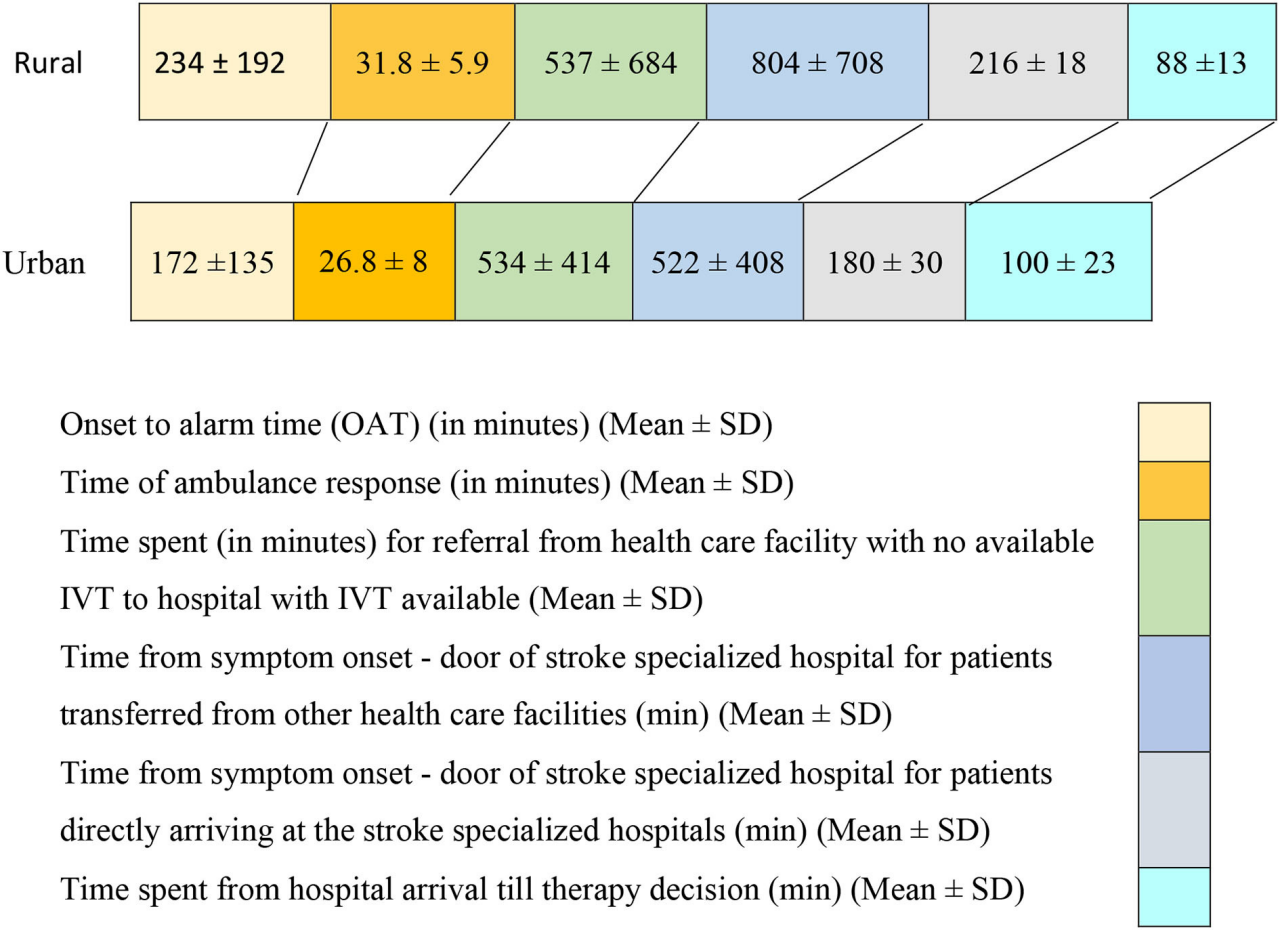
Time metrics variations among rural and urban patients.

Notably, 81.9% of patients coming from rural regions were first treated by non-stroke-ready hospitals and needed referral to an IVT-capable center (Table 1). The time needed to refer a patient from the hospital where IVT was not available to the hospital where IVT was available did not differ between rural and urban regions (537 ± 684 min in rural and 534 ± 414 min in urban patients) (Figure 3). In patients, who arrived at one of the three stroke centers within their IVT treatment window, the time from symptom onset to the stroke center door was significantly longer for patients coming from rural areas (mean 216 ± 18 min) compared to patients coming from urban areas (mean 180 ± 30 min). This inequity was similar for patients who arrived at the stroke hospitals outside the IVT time window. The patients coming from rural areas needed (804 ± 708 min) from symptom onset to the stroke center door, which was significantly longer than for those who came from urban areas (mean 522 ± 408 min) (Table 4, Figure 3). Notably, 73.3% of patients arriving at the hospital within the IVT time window came from urban regions. Approximately 63.6% of urban and 59% of rural patients arriving at the hospital where IVT was available within the time window were men; however, there was no statistically significant difference in sex (Table 4). Among patients presenting to hospitals within the therapeutic time window, 86.4% of the rural patients presented within 3.5–4 h from stroke onset, which left only a narrow therapeutic time window (Table 4). Time spent in imaging for rural patients with stroke was 64.7 ± 12.3 min, which is lower than for urban patients with stroke 75.1 ± 24.5 min. 29.8 % of urban patients with stroke had MRI imaging for their stroke diagnosis in comparison to 11.4% only of rural patients with stroke. Surprisingly, patients with stroke who were eligible for thrombolysis but did not receive it had a door to stroke unit management of 100.1 ± 23.5 min if coming from urban regions, which was significantly higher than for rural patients with stroke (88.4 ± 13.8 min; Table 4).

**Table 4:** General demographic characteristics of patients arrived within 4.5 h after symptom onset.

	**Rural** **(*n* = 44, 12%)**	**Urban** **(*n* = 121, 47.6 %)**	***p*-value**
Number patients arrived within time window (%)			<0.05
Within 2 h of stroke onset	1 (2.3%)	14 (11.6 %)	
Within 2.5 h of stroke onset	5 (11.4%)	16 (13.2%)	
Within 3 h of stroke onset	16 (36.4 %)	47 (38.8%)	
Within 3.5 h of stroke onset	22 (50 %)	38 (31.4%)	
Within 4 h of stroke onset		6 (5%)	
Male (*n*, %)	26 (59%)	77 (63.6%)	0.09
Females (*n*, %)	18 (41%)	44 (36.4%)	
Time from symptom onset - door of stroke specialized hospital for patients arrived outside IVT time window (min) (Mean ± SD)	804 ± 708	522 ± 408	<0.05
Time from symptom onset - door of stroke specialized hospital for patients arrived within IVT time window (min) (Mean ± SD)	216 ± 18	180 ± 30	
Time from hospital arrival- meeting neurologist (min) (Mean ± SD)	12.7 ± 2.7	12.6 ± 2.8	0.86
Time spent for laboratory investigations in minutes (Mean ± SD)	97.9 ± 59.3	121.3 ± 74.1	0.06
Time spent for imaging in minutes (Mean ± SD)	64.6 ± 12.2	75.12 ± 24.45	<0.05
Type of first imaging (*n*, %)			<0.05
CT	41 (93.2%)	89 (73.6%)	
MRI	3 (6.8%)	32 (26.4%	
Time spent from hospital arrival till therapy decision (min) (Mean ± SD)	88.4 ±13	100.08 ± 23	<0.05

Data described as numbers and percentages or mean ± standard deviation according to need. Q square and independent *t-*tests were used.

## Discussion

Acute stroke management and IVT utilization showed progress in Egypt. Despite that, IVT is still far away from the aimed rate (9, 10). In this study, we studied the main causes of delayed presentation to hospitals and in-hospital obstacles to the administration of IVT among urban and rural Egyptian patients with stroke.

The current study has shown that 47.6% of urban patients arrived within the therapeutic time window in comparison to 12% only of rural patients. Our results were in line with another report that showed pre-hospital delay is more common among rural patients; however, no definite proportion of rural patients with stroke presenting outside the time window in comparison to urban patients was reported (9). Another study showed a proportion as low as 26.4% of urban patients arriving at the hospital within the time window. These results were lower than our results, which may be attributed to the conduction of this study in 2015 when IVT was not widely available in Egypt and not sponsored by the Egyptian government (10).

A recent study examining the main causes of pre-hospital delays in EDs found that living in rural areas is one of the main causes of delayed presentation (14). Similar results were obtained in a cohort analysis conducted in the southwest of Germany. Compared to rural patients, patients living in urban areas were more likely to present themselves within the time window (15). This rural–urban gap also exists in high-income countries (16, 17).

The ODT was 804 ± 708 min among our rural patients who arrived outside the time window, which was significantly higher than for urban patients (522 ± 408 min). The reported data from Egypt regarding ODT showed marked heterogeneity; one study showed that ODT was 162 min (18). This significant difference is attributed to a difference in study participant selection criteria, which excluded patients with acute stroke presenting later than 24 h after symptom onset. Our results were shorter than another Egyptian study (19), which reported that was 1,956 ± 1,968 min. Our results were similar to the reports from other countries with ODT among rural patients ranging from 300 to 1,800 min (20–23). The ODT for patients who arrived within the therapeutic time window was 216 ± 18 min among rural patients, which was also significantly higher than for people living in urban regions (180 ± 30 min). This means that even when rural patients arrived within the therapeutic time window, they have limited chance to take the thrombolytic therapy due to narrow in-hospital time.

This delay in ODT could be attributed to multiple factors. A major factor is a delay in help seeking. Onset to alarm times was nearly 4 h among rural patients, which was significantly higher than the 3 h patients from urban areas needed to call. This onset to alarm times was longer than the results described in the literature (20, 24). This delay in onset to alarm times could be explained by the low level of stroke awareness among the Egyptian population (25). Patients with stroke in rural regions tended to alarm ambulances (26.6% of patients), general practitioners (GPs) or internal medicine doctors, relatives, neighbors, neurologists, and other medical specialties. While urban patients alarmed ambulances (20.1%), neurologists, relatives, neighbors, internal medicine doctors, GPs, and other specialties. A recent Egyptian study (10) identified that nearly 20% of urban patients in Egypt used ambulances to reach the hospital. Similar to our study patients tended to alarm neurologists, internists, GPs, and other specialties. Also, data coming from the Netherlands showed that patients alerted relatives or friends, GPs, and ambulances (26). The pre-hospital delay in our study may be attributed to the fact that more than half of our patients were referred from non-stroke-ready hospitals, private hospitals, or community clinics. The majority of referred patients (88%) were rural patients, which likely is caused by a major deficit of stroke-ready hospitals in rural Egypt. Pre-hospital delays of rural patients are not a unique phenomenon in low-middle-income countries, but also reported in high-income countries, for example, in the United States, data from the national stroke registry has consistently related the arrival to a rural hospital as one of the factors to the failure of IVT (27). Ambulance response time to patients with stroke was significantly longer in rural regions compared to urban regions. This delayed response time does not meet the established American Heart Association/American Stroke Association guidelines for being at the scene within <15 min (28). Despite this delay, it is not far from the time recorded in other high-income countries like the United States (29). Pre-hospital delays in rural Egyptian areas can also be explained by the presence of the Egyptian ambulance system solely focused on urban regions. Misdiagnosis of stroke symptoms is another factor adding to delays. In our study, most patients were not identified as suspected strokes (rural patients 69%, urban patients 56%). This can be explained by false referrals of the patients to non-stroke specialists. Another reason might be that patients themselves directly went to seek help from non-stroke specialists in private hospitals after misjudging their symptoms. This percentage was much higher than those recorded by other Egyptian authors (10), who reported that the stroke misdiagnosis by the first examination was 18.2%. In our study, only 8.2% of rural patients and 19.7% of urban patients were initially examined by neurologists compared to 44.2% in other studies (10). Our results were lower than another Egyptian study (19), which found that 82.8% of patients with stroke were initially misdiagnosed by the first examiners. Surprisingly, the door to decision of therapy among urban patients was significantly longer than among rural patients (100 ± 23.4 min) vs. (88.40 ± 13.79 min), respectively. This difference could be explained by 26.4% of urban patients with stroke who had brain MRI to diagnose their stroke in comparison to only 6.8% of rural patients, which represents an important factor for in-hospital delays in urban patients. Our results were similar to Abraham and collaborators (30), who found that the door to decision in tertiary care centers in India was 94.17 ± 54.5 min.

Delays in laboratory investigations and imaging durations were the most common causes of in-hospital delays in our study. Laboratory investigations done routinely in patients with stroke prior to treatment were based on the high number of patients affected by the hepatitis C virus (31).

An earlier Egyptian study found that the unavailability of the IVT drug, lack of physician experience, and imaging delays were the most common causes of in-hospital delay (10). Their study was performed before IVT was widely available and covered by the government. Our results were far away from the standards of international recommendations for door to decision of management times, especially in rural areas, and this is a call for health authorities to invest in health education and acute stroke management program.

Our study has several limitations. We did not investigate the patient's education, level of stroke awareness, and economic level as the possible causes of the pre-hospital delays. We did not investigate the time lost in administrative issues as a possible cause of in-hospital delays. The demographic data analysis shows a much greater proportion of male participants so these results might vary among women with acute stroke.

## Conclusion

Increasing population awareness about early stroke identification and the value of early arrival to stroke-ready hospitals, and training EMS personnel about bypassing non-stroke-ready hospitals to the nearest ready one, could reduce pre-hospital delays. The establishment of more stroke-ready hospitals and training of more physicians on acute stroke management in rural Egypt would reduce the burden on the urban hospitals and improve stroke systems of care among rural patients.

## Data availability statement

The raw data supporting the conclusions of this article will be made available by the authors, without undue reservation.

## Ethics statement

The studies involving human participants were reviewed and approved by Ethical Committee of Faculty of Medicine, Assuit University. The patients/participants provided their written informed consent to participate in this study.

## Author contributions

AN: conceptualization, methodology, and writing the manuscript. SW: supervision and writing and reviewing. KM, AG, and AD: data collection. GS: conceptualization and data collection. KF: supervision and project administration. FA-A: conceptualization and writing the manuscript and reviewing. All authors have read and approved the manuscript.
