# Supplementary material for: Pre- and in-hospital delays in the use of thrombolytic therapy for patients with acute ischemic stroke in rural and urban Egypt
Source: Front Neurol. 2023 Jan 20;13:1070523. doi: 10.3389/fneur.2022.1070523 (PMC9895407; doi:10.3389/fneur.2022.1070523)
Supplement: Supplementary file 1 [file Table_1.DOCX]

**Patient Form**

**Demographic:**

Patient Number: Patient initials: Date:

Age: Sex: rural/ urban:

**Setting:**

Emergency site:

1. Urban setting:

2. Mixed urban and rural setting:

3. Rural setting:

Clinic:

1. Assuit:

2. Cairo:

3. Tanta

Distance to the hospital:

Duration of getting to the hospital:

**Medical data**

- Risk Factor:
- Which first symptoms:
- Initial (suspected) diagnosis:
- Final/ discharge diagnosis:
- mRS at admission:
- mRS at discharge
- NIHSS at admission:
- NIHSS at discharge

**Process management**

- Time of onset of first symptoms (8am -3pm, 3pm-10pm,10pm-8am)
- Time from first symptom occurrence till alarm
- Which alarm the patient notified when he had stroke
- Time of ambulance response
- Method of transport to the hospital if the relatives did not call the ambulance
- If patients referred from one hospital to another where rtPA is available
- Time wasting in referral from one hospital to another
- Time of alarm of EMS or other (which) persons (if any)
- Time of EMS arrival (if any)
- Means of transport to hospital, e.g., private transportation (which), EMS, other:
- Time of hospital arrival:
- Time between symptom onset to hospital door:

**Patients arrived hospital within time window but did not receive rtPA**

- Time from hospital door to neurological examination:
- Time till laboratory results delivery:
- Time spent in imaging:
- Which imaging the patients had
- Time spent between first arrival to the hospital and taking the decision of therapy
